# Experiences of primary healthcare professionals and patients from an area of urban disadvantage: a qualitative study.

**DOI:** 10.3399/bjgpopen19X101676

**Published:** 2019-11-27

**Authors:** Jennifer Reath, Marlee King, Walter Kmet, Diana O'Halloran, Ronald Brooker, Diana Aspinall, Hani Bittar, Thava Seelan, Michael Burke, Tim Usherwood

**Affiliations:** 1 Professor, School of Medicine, Western Sydney University, Sydney, Australia; 2 Sessional Academic, School of Psychology, Western Sydney University, Sydney, Australia; 3 Chief Executive Officer, Macquarie University Hospital and Clincial Service, Macquarie University, Sydney, Australia; 4 Conjoint Associate Professor, Western Sydney University, Sydney, Australia; 5 Conjoint Professor, School of Medicine, Western Sydney University, Sydney, Australia; 6 Chair, WentWest Ltd, Sydney, Australia; 7 Research Affiliate, School of Medicine, Western Sydney University, Sydney, Australia; 8 Consumer Representative, School of Medicine, Western Sydney University, Sydney, Australia; 9 General Practitioner and Conjoint Lecturer, School of Medicine, Western Sydney University, Sydney, Australia; 10 General Practitioner and Conjoint Lecturer, School of Medicine, Western Sydney University, Sydney, Australia; 11 General Practitioner and Conjoint Associate Professor, School of Medicine, Western Sydney University, Sydney, Australia; 12 Professor of General Practice and Head of Westmead Clinical School, The University of Sydney, Sydney, Australia

**Keywords:** Primary health care, general practitioners, socioeconomic factors, delivery of healthcare, qualitative research

## Abstract

**Background:**

The health disadvantage in socioeconomically marginalised urban settings can be challenging for health professionals, but strong primary health care improves health equity and outcomes.

**Aim:**

To understand challenges and identify needs in general practices in a socioeconomically marginalised Australian setting.

**Design & setting:**

Qualitative methodology with general practices in a disadvantaged area of western Sydney.

**Method:**

Semi-structured interviews with healthcare professionals and their patients were transcribed and analysed thematically under the guidance of a reference group of stakeholder representatives.

**Results:**

A total of 57 participants from 17 practices (comprising 16 GPs, five GP registrars [GPRs], 15 practice staff, 10 patients, and 11 allied health professionals [AHPs]), provided a rich description of local communities and patients, and highlighted areas of satisfaction and challenges of providing high quality health care in this setting. Interviewees identified issues with health systems impacting on patients and healthcare professionals, and recommended healthcare reform. Team-based, patient-centred models of primary health care with remuneration for quality of care rather than patient throughput were strongly advocated, along with strategies to improve patient access to specialist care.

**Conclusion:**

The needs of healthcare professionals and patients working and living in urban areas of disadvantage are not adequately addressed by the Australian health system. The authors recommend the implementation of local trials aimed at improving primary health care in areas of need, and wider health system reform in order to improve the health of those at socioeconomic and health disadvantage.

## How this fits in

Access to health care is often inadequate in areas of socioeconomic and health disadvantage. While primary health care is critical in such settings, they can be challenging places to work.

This research describes the experience of primary healthcare professionals in a socioeconomically disadvantaged urban area in Australia. Interviewees report that the Australian healthcare model fails to adequately address health needs in this setting or reward quality care, and recommend systems change to address this.

## Introduction

Australia struggles to provide equitable access to high quality health care across its population.^1^ Much focus to date has been on rural regions, often sparsely populated and at considerable distance from health care. While these communities are important to consider, in Australia as elsewhere, immigration and population drift from rural to urban areas as a result of the search for employment have contributed to growing urban communities at socioeconomic and health disadvantage.^2^

For many living in such settings, health disadvantage and underlying social determinants of health impact on access to health care. High rates of disability as a result of chronic diseases, compounded by cost and transport constraints, social exclusion, different language and cultural needs, mental health problems, and poor health literacy create difficulties in accessing and navigating complex health systems.^3,4^ High quality primary healthcare is critical in disadvantaged urban settings, yet the challenges of working in such settings threaten the sustainability of the workforce.^5^

The south west area of Blacktown is a socioeconomically disadvantaged area in western Sydney. According to the Index of Relative Socio-economic Disadvantage (IRSD; an index derived from census data related to income, education, and employment), the south west area of Blacktown, where the present research focused, includes areas such as Mt Druitt, with IRSD scores as low as 480, compared with the average Australian score of 1000. The health risks and diseases prevalent in this area reflect this disadvantage, with high rates of smoking and obesity, heart disease, asthma, diabetes, and poor mental health. The population includes many migrants, refugees, and Aboriginal people.^6^

Australian GPs are generally self-employed, with income derived predominantly through the Australian government-funded Medical Benefits Scheme (MBS) according to time spent with patients. Patients are not registered to a particular practice. Team-based primary health care is growing in Australia, with many practices employing nurses to provide chronic disease and preventive health care. Australia is among Organisation for Economic Cooperation and Development countries, with the highest proportion of GP income derived from fee-for-service payment.^7^ Most specialist care is provided by private practitioners who often charge fees in excess of the MBS patient reimbursement.

The needs of healthcare professionals must be considered in health service and workforce planning in order for equitable access to health care and improved health outcomes to be achieved. The aim of the research reported in this article was to explore the experience of GPs, other primary healthcare professionals, and patients, living and working in a marginalised area of Sydney.^8^

## Method

Data was collected between March 2015–March 2016 using a qualitative approach. Semi-structured interviews were conducted to explore the experience of primary healthcare professionals and their patients in the Blacktown area, with a particular focus on those in disadvantaged postcodes. The authors worked with WentWest Ltd. This is the Western Sydney Primary Health Network (previously Western Sydney Medicare Local), one of 31 regional organisations funded by the Australian government to support primary care. With their assistance, the authors recruited a reference group, including three GPs, a practice nurse, three WentWest senior managers, the Chair of the WentWest board, and a patient representative.

The authors developed a semi-structured healthcare professional interview schedule in consultation with the reference group and, guided by the patient representative, adapted this for patients. WentWest invited GPs known to practice in disadvantaged areas of Blacktown to participate in the study. Recruited GPs invited practice staff, AHPs, and patients, and were encouraged to promote the research to other GPs in their vicinity. The authors maximised the variety of participants across practice types (solo, group, privately owned, and corporate practices) and AHP types by directly approaching lead GPs in underrepresented practice types.

All participants provided informed, written consent and interviews were conducted by a single researcher either face-to-face or by telephone, according to participant preference. Interviews were mostly of 30–45 minutes duration, with the longest lasting 1 hour and 13 minutes, and the shortest just over 14 minutes. Interviews were recorded and transcribed verbatim by an independent transcription service, integrity checked, and de-identified. Recruitment was continued until the reference group was satisfied that a range of practitioners across a variety of practice types had been interviewed and analysis indicated thematic saturation.

Transcripts were analysed thematically^9^ in parallel with data collection, using NVivo (version 10) to organise the data. An early coding framework was discussed with the reference group, and newly emerging themes were considered by the research team and finalised in discussion with the reference group. These discussions enabled the authors to reflexively consider how their own experiences and backgrounds influenced their interpretation of the data in accordance with recommendations for reporting qualitative research.^10^

## Results

The authors interviewed 57 participants associated with 17 general practices in the targeted area, comprising 16 GPs, five GPRs, eight receptionists, seven practice nurses, 10 patients, and 11 AHPs. One GP had moved out of Blacktown and contacted the authors following an approach by the GP’s previous practice receptionist. Emergent codes were aggregated under four overarching themes: perceptions of local communities and patients; attitudes of healthcare professionals to their work; structural issues impacting on general practice; and concerns about health systems. The codes related to each theme are described below, with representative quotations presented in Box 1.

Box 1 **Themes and illustrative quotations**

**Table:** 

Themes and codes	Illustrative quotations
**Perceptions of local communities and patients**	
**Cultural hub**	*'* *We are seeing a lot of newly arrived migrants and refugees and recently we start to see a lot of refugees lately* *. '* (AHP01)
**Socioeconomic and health disadvantage**	*'* [We] *do get a fair amount of mental health, anxiety, depression in the teenagers … In the middle age sort of category, again a lot of anxiety, a lot of stress-related depression* *. '* (GP06) *'* *Chronic disease is pretty much prevalent in this area* *…* *Western Sydney is a hot spot for diabetes* *…* *'* (GP01)
**Patient attitudes to health**	*'* *… because of the poor education … they don’t have the understanding to look after themselves.* *'* (GPR05) *'* *…* [patients] *can afford to spend money on the cigarettes but … have no money to spend on medications.* *'* (GP12). *'* *When they know about their medicines they are going to be empowered to … grab hold of their own situation. I find that when they don’t know what they’re taking things for* *… they’re lost* *… just taking things but they’re not taking responsibility for their own health* *. '* (AHP04)
**Attitudes of healthcare providers to their work**	
**Commitment to high quality health care**	*'* *…* *People come in with mental health issues and I don't know if you can do that in six minutes* *…* *I educate my patients about why they can't have antibiotics for a cold, I take the time to do that* *.* *'* (GP06) *'* *… if he thinks there’s really something wrong, he’ll do everything to find out what it is* *… if you’re really sick, he will go out of his way to try and fix you up* *…* *I like that.* *'* (Pt04)
**Importance of trust and strong relationships with patients**	*'* *So they* *…* *adopt you into their lives and you become part of their lives* *…* *and they think of you as a sister or a daughter, or a surrogate mother* *. '* (GP04) *'* *You kind of have to relate to them really and explain to them why from a medical perspective X, Y, Z is indicated or not indicated, and ask why they believe what they believe, and then if they tell you, then you can discuss it. But if they don’t feel that they can relate to you … then the trust and therapeutic relationship is not working.* *'* (GP15)
**Enjoy the work**	*'* *I have a lot of professional satisfaction from working here. So I love my job and I love the staff and I love my patients* *.'* (GP08) *'* *I just I enjoy the fact that the things that we’re implementing actually make a change and this person manages their health, they don’t end up going to hospital every other week* *. '* (PN03). *'* *… I prefer not to* [work] *in affluent areas* *…* *there are obviously illnesses everywhere, but I do feel a bit more useful in the area that’s less affluent.* *'* (GP14)
**Challenging nature of the work**	*'* *It’s exhausting and it’s one of those jobs that you’re working pretty much from the minute you get out of bed until the minute you go back to bed* *…* *'* (Rct02) *'* *We don’t have enough time to give to the patient to really go deeply into the problems* *… putting band aids on it, which I don’t like.* *'* (GP11) *'* *Mental health* *— it does eat into your time a lot. They'll come in with a 15 minute appointment* *… 20 minutes later you've already eaten up 35 minutes and it makes you late. That does increase my stress.* *'* (GP06) *'* *… you'd like to think that there's a specialist who can help you take care of the patient* *…* *but if this patient won't go in to see them* *…* *it keeps you awake at night.* *'* (GPR04) *'* *…* *patients just don’t realise that he’s only one doctor and there’s just not enough time* *…* *'* (Rct05) *'* *I think it takes its toll* *… medicine has one of the highest divorce rates and one of the highest suicide rates out of the professions* *… often it ends up in situations where there’s … burnout* *… people just can’t* *… cope with that.* *'* (GP09)
**Structural issues impacting on general practice**	
**Impediments to quality care**	*'* *…* *I've seen so many* [doctors] *which makes it hard because then they say,* *"* *Have you had this vaccination?* *"* *And I think, "I can't remember and it's probably not on your record"* *. '* (Pt02)
**Value of team-based approach**	*'* *…* *I do the dressing, they’re not sitting out there waiting half an hour for the doctor* *. '* (PN03) *'* *I like being part of the team. It’s better than working in isolation.* *'* (AHP04) *'* *… you need the right support staff* *… I'm not a dietician, I'm not a psychologist, I'm not a physio* *…* *I'll use some of those elements of those in my normal day-to-day routine* *…* *'* (GP10) *'* *When you’re sick, virtually you have to close the practice, because you can’t have somebody else come in* *…* *'* (GP09) *'* *We are really lucky to have Mt Druitt Association … that relationship with other doctors and we can talk about things.* *'* (GP14)
**Health systems concerns**	
**Importance of primary health care**	*'* *…* *governments don’t appreciate general practice …* *governments just say,* *"* *… we’ll just turn the screws a little bit,* [GPs] *won’t mind* *"* *.* *'* (GP09) *'* *I see patients around every 10 minutes or 15 minutes* *…* *process information* *… put a plan and I need to make … sometimes two or three decisions in the same consultation* *… all your decisions has medical and legal consequences* *… for your consultation you’re going to get $35* [AUD] *and you pay at least 40% overheads* *… leave you with around $20. Then you pay tax* *…* *that’s $10. So you study 13 years, you make a lot of decisions* *…* *for ten bucks. Not fair I think* *.* *'* (GP08)
**Fragmented health system**	*'* *Trying to get blood test results from the hospital was a time-consuming and quite honestly frustrating process* *… So there’s the hospital’s existence, silos, whereas we are trying to do the opposite* *.* *'* (GP05) *'* *It is not good enough for individual patients to have isolated experiences of excellent care. They actually need care which is stitched up* *. '* (GP07)
**Barriers to accessing healthcare**	*'* *… they’re in crisis I find it’s very, very difficult to get them engaged with the services …* *contact an access team or a mental health crisis team. They’ve got all of these different red tapes and criteria* *…* *'* (PN07) *'* *…* *had to wait a month just to do the consultation with the specialist, a few months more for the surgery* *… through the public hospital system* *. '* (Pt07) *'* *… can’t afford medication, they have no transport, they have no money for fuel even when they have a car or it’s not registered or when I refer them to see a specialist or to other health professionals they can’t afford to pay* *.'* (GP12)
**Difficulty navigating the health system**	*'* *…* *the health system, it’s pretty complex for me to do and I’ve been working for New South Wales Health for 18 years* *…* *So for someone who is not well educated who has lots of pressures, they haven’t got a chance really to find out what help there is for them.* *'* (PN03)
**Funding systems**	*'* *…* *doctors are often quite willing to donate a bit of time but especially when we’re talking about* *…* [AHPs]*, their hourly rate is not actually that high so it is quite a big ask to ask them to donate more time* *. '* (GP05) *'* *…* *you have the Federal who looking after Medicare and you have State who looking after hospital* *… a lot of improvement needs to be done.* *'* (GP08) *'* *…* *where I'm concerned is health should be funded by one body.* *'* (GP13)
**PHN support and call for advocacy**	*'* *They're* [PHN] *just so supportive* *… having that number to ring and say,* *"* *I don't know* *"* *… And they were the ones that sort of* *got me on to a few different programs* *…* *got me orientated* *. '* (PN01) *'* *…* *put this research* *… in front of the media … doctors are very busy, they don’t talk. And other people are talking a lot, very loud* *…* *we cannot do it* *. '* (GP11)

AHP = allied health professional. GPR = general practice registrar. PHN = primary health network. PN = practice nurse. Pt = patient. Rct = receptionist.

### Perceptions of local communities and patients

Interviewees described their communities as 'cultural hubs' with many migrants and language groups, and growing numbers of refugees. Though some areas of affluence were noted, high levels of socioeconomic disadvantage — closely intertwined with poor physical and mental health, and a growing prevalence of chronic illness and multimorbidity — were highlighted as major challenges.

Financial barriers prevented many patients attending private specialists, and GPs struggled to provide high quality clinical care without this support. GP interviewees described the time spent identifying affordable options. Some healthcare professionals commented on patients lacking motivation to change unhealthy behaviours, while others noted that not all fitted this stereotype. A number of interviewees recognised the importance of education to improve health literacy and identified patient empowerment as critical in improving health.

### Attitudes of healthcare professionals to their work

In spite of challenges in providing health care in this setting, most interviewees highlighted their commitment to providing high quality care, often expressing this as an ethical imperative, and care was underpinned by long-standing, empathetic relationships. Time was required within consultations to develop these relationships and to provide quality care, which was highly valued by the patients interviewed.

Many healthcare professionals enjoyed the challenges of their work and the opportunity to make a difference in an area of disadvantage. However, not all shared this view, and the one GP who had left the area explained that this was due to limited resources and a lack of patient compliance. Others described their emotional and physical exhaustion, and difficulties addressing patient needs, especially mental health needs, with limited time in the consultation.

Other difficulties reported included the high prevalence of disease (particularly diabetes), unrealistic patient expectations, and the challenge of trying to address patients’ physical, psychological, and social needs. For some, notably GPRs, the stress of managing patients who could not afford specialist medical care kept them *'* *awake at night* *'* (GPR04). The impact of work on personal lives and emotional wellbeing was described as a risk for burnout.

### Structural issues impacting on general practice

All interviewee groups commented extensively on structural impediments to quality patient care, including inflexible appointment systems and long waiting times. These were said to result in patients visiting high turnover medical centres for minor health problems, rather than their regular general practice. Both GP and patient interviewees highlighted the disadvantages of having multiple healthcare professionals.

Working as a team in primary health care was seen by professionals and practice staff as critical to managing patient needs, improving practice efficiency, and enhancing health professional satisfaction, yet the lack of funding support for team-based care, including for practice nurse employment and for AHP input to care planning, was described as a barrier that needed to be addressed.

Although most solo GPs noted advantages of this practice style, describing strong intergenerational relationships with patients, many interviewees described the solo GP as *'* *a dying breed* *'* (GP13). Solo GPs spoke of isolation and challenges recruiting additional GPs and locums. They valued the support the Primary Health Network (PHN) provided in arranging after-hours patient care, as well as the support provided through the Mt Druitt Medical Practitioners Association.

### Health systems concerns

Most health professional interviewees emphasised the importance of primary health care, particularly preventive care. They highlighted potential cost savings through avoidance of hospitalisation. Some considered that general practice was undervalued by *‘* *the government* *’* (GP09). The Australian fee-for-service model was described as providing inadequate payment and encouraging patient throughput rather than quality practice.

Many interviewees commented on fragmentation of the health system, with poor communication between general practices, hospitals, and other providers causing concern particularly in relation to hospital admission and discharge, and provision of information about medications and pathology results. The choice of some patients to see multiple GPs and lack of connection between general practices was also seen as problematic. There was a strong call for better integration of health care with a range of strategies proposed, including shared electronic records, hospital liaison staff, and patient registration with a single general practice.

Patients and health professionals described barriers to accessing appropriate healthcare, including bureaucratic hurdles and wait times for public services, as well as costs of accessing private specialist care. Even the costs of medications and maintaining a healthy lifestyle were reported to be beyond the means of many patients. Working with patients to overcome these challenges imposed additional workload on healthcare providers. Both patients and GPs valued specialists and AHPs who were willing to bill only the Medicare rebated fee.

Another barrier to access was difficulty with navigating complex health systems. Health Pathways, an online guide to local services, was reported to assist in addressing this concern, although the need for ongoing maintenance of this resource was seen as a risk.

A number of interviewees commented on challenges associated with health funding coming from two levels of government — public hospitals funded by state governments, with general practice and other private providers reimbursed wholly or partly under the Australian Government Medicare programme.

While frustration with health systems was a strong theme in interviews, so too was the valuing of support provided by the local PHN, including information technology assistance, practitioner education, assistance with access to patient psychological services, and support for practices transitioning to Patient Centred Medical Home (PCMH) models of practice.

Finally, GP interviewees wanted the important role of general practice better promoted, and for this research to be used to advocate for changes in the health system, in order to improve support for high quality primary health care.

## Discussion

### Summary

Primary healthcare professional and patient interviewees described western Sydney as culturally diverse with high rates of socioeconomic disadvantage, closely intertwined with poor physical and mental health. They discussed challenges of managing complex health problems when patients with limited health literacy, and often speaking English as a second language, were unable to access expensive specialist care. Health professional interviewees set a high priority on providing high quality care, regardless of time and financial constraints, and valued longstanding, trusting relationships. They described the toll this work took and called for greater valuing of primary healthcare professionals working in disadvantaged communities. Multidisciplinary, team-based care aligning with PCMH models was recommended, with patient enrolment and improved integration between health sectors. To enable these new models of care, interviewees proposed a change from the fee-for-service model that rewards patient throughput rather than quality care.

### Strengths and limitations

A key strength of this research was the opportunity provided by semi-structured interviews to explore the experiences of those interviewed. While findings accord with other studies of health care in areas of socioeconomic and health disadvantage, the authors may have undersampled those more taxed by their work and less engaged with the university and the PHN, as well as those less committed to working in socioeconomically disadvantaged areas. Inclusion of the perspective of one GP who had left their practice in the targeted area provided a negative counterpoint to other views expressed; however, other views described may be overly optimistic.

### Comparison with existing literature

The association between low socioeconomic status and higher levels of ill-health is well described.^11–13^ Similarly, poorer access to health care for those in most need of that care is not a recent finding.^14,15^ Australian general practice data indicate that socioeconomically disadvantaged patients have fewer preventive health consultations, more chronic health problems, and lower rates of referral for pathology and to specialists.^16^ Socioeconomic disadvantage has been shown in the UK to influence referral practice and healthcare navigation, which is more complex in disadvantaged areas.^17^ Additionally, in the Australian context, those with chronic disease, especially mental health conditions, are more likely to forgo health care as a result of high out-of-pocket costs.^18^

The challenges described by GP interviewees are similar to those experienced by GPs in disadvantaged areas of Scotland, where GPs have been described as working in the 'Deep End' — struggling to meet health and various other needs in their communities.^5^ More recently, a study exploring resilience of GPs working in a deprived area of England described the integration of personal and professional values and the importance of supportive teams, both highlighted by interviewees within the present study.^19^ Strategies described as having been used by family physicians working in disadvantaged communities in Montreal^20^ are also similar to those referred to by interviewees of the present study: building personal relationships with patients; seeking to understand their patients’ experiences of disadvantage and its impact on their health; and working in teams to empower patients. The empathetic, longstanding relationships valued in the current study by both healthcare providers and patients have been shown in earlier work to have a significant impact on patient enablement, including in deprived areas.^21^

A recent Australian report investigating 'hotspots' of health inequality resulting in high rates of potentially preventable hospital admissions has recommended trials of local, tailored, rigorously evaluated approaches to reducing health inequalities, with the aim of reducing preventable hospitalisations and resulting health costs.^1^ Recent work in western Sydney implementing PCMH models^22^ and supporting integration of health services in this region^23^ exemplifies such approaches. In a similar setting of deprivation in Scotland, a 'non-geographic cluster' of GPs provided with protected time to work collaboratively on service and professional development has resulted in high levels of satisfaction for the GPs.^24^ This model incorporates many elements of a similar quality improvement intervention shown at 12 months to significantly improve several indicators of patient wellbeing, resulting in an estimated cost-effectiveness ratio of 12 224 GBP per quality-adjusted life year gained.^25^

Beyond such local interventions, many of those interviewed in the present study called for systemic transformation strengthening primary health care, particularly through funding models rewarding quality rather than occasions of care. Other recommendations for improving primary health care included patient enrolment with a single practice, and enhanced support for team-based care. Given the association between strong primary care and improved and more equitable health outcomes,^26,27^ approaches strengthening the role of primary health care would likely assist in addressing health inequity in settings such as western Sydney.

Interestingly, given the challenges noted with fee-for-service payment by those interviewed for this study, a recent UK publication notes challenges also with capitation funding of primary health care in areas of socioeconomic disadvantage.^28^ Addressing both the concerns of the present study’s interviewees and those of the UK research, a recently published commentary in the US highlights the importance of freeing primary care physicians up from maximising throughput, and improving support for multidisciplinary team-based care, in order to improve the management of the sorts of complex patient problems described by GPs in the present study.^29^ Many of Goroll’s recommendations, as well as those of the present study’s GPs, conform with PCMH models, which are increasingly evidenced as improving health outcomes.^30^ The Australian government has recently funded a national trial of a PCMH-related model of care (the Health Care Home);^31^ however, GPs in western Sydney have expressed concern that this model is limited in its scope and is underfunded, and therefore unlikely to influence health outcomes.^22^

This study’s interviewees called for improved access to specialist and other health services for those unable to afford this health care. The Closing the Gap programme which provides Aboriginal and Torres Strait Islander patients with top-up payments for non-GP specialist care, as well as additional funded AHP visits and support for purchase of pharmaceuticals, may provide a model for prioritising those most in need of improved access.^32^

### Implications for research and practice

The insights from the present research into the needs of patients and healthcare professionals in a marginalised urban setting in Australia provide a strong call for transformation of Australian primary health care. The authors recommend local trials that are rigorously evaluated to investigate the impact of new models of care in areas of need, and consideration of wider health system reform to better address the needs of those at socioeconomic and health disadvantage.
